# Space-Time Clustering Characteristics of Tuberculosis in China, 2005-2011

**DOI:** 10.1371/journal.pone.0083605

**Published:** 2013-12-19

**Authors:** Fei Zhao, Shiming Cheng, Guangxue He, Fei Huang, Hui Zhang, Biao Xu, Tonderayi C. Murimwa, Jun Cheng, Dongmei Hu, Lixia Wang

**Affiliations:** 1 National Center for Tuberculosis Control and Prevention, Chinese Center for Disease Control and Prevention, Beijing, China; 2 School of Public Health, Fudan University, Shanghai, China; 3 AIDS & Tuberculosis Unit, Ministry of Health and Child Welfare, Harare, Zimbabwe; Arizona State University, United States of America

## Abstract

**Objectives:**

China is one of the 22 tuberculosis (TB) high-burden countries in the world. As TB is a major public health problem in China, spatial analysis could be applied to detect geographic distribution of TB clusters for targeted intervention on TB epidemics.

**Methods:**

Spatial analysis was applied for detecting TB clusters on county-based TB notification data in the national notifiable infectious disease case reporting surveillance system from 2005 to 2011. Two indicators of TB epidemic were used including new sputum smear-positive (SS+) notification rate and total TB notification rate. Global Moran’s *I* by ArcGIS was used to assess whether TB clustering and its trend were significant. SaTScan software that used the retrospective space-time analysis and Possion probability model was utilized to identify geographic areas and time period of potential clusters with notification rates on county-level from 2005 to 2011.

**Results:**

Two indicators of TB notification had presented significant spatial autocorrelation globally each year (*p*<0.01). Global Moran’s *I* of total TB notification rate had positive trend as time went by (*t*=6.87, *p*<0.01). The most likely clusters of two indicators had similar spatial distribution and size in the south-central regions of China from 2006 to 2008, and the secondary clusters in two regions: northeastern China and western China. Besides, the secondary clusters of total TB notification rate had two more large clustering centers in Inner Mongolia, Gansu and Qinghai provinces and several smaller clusters in Shanxi, Henan, Hebei and Jiangsu provinces.

**Conclusion:**

The total TB notification cases clustered significantly in some special areas each year and the clusters trended to aggregate with time. The most-likely and secondary clusters that overlapped among two TB indicators had higher TB burden and risks of TB transmission. These were the focused geographic areas where TB control efforts should be prioritized.

## Introduction

Great achievements have been made for Tuberculosis (TB) control during last two decades. The prevalence of TB had decreased globally from over 250 cases per 100,000 population in 1990 to 170 cases per 100,000 in 2011[1]. However, TB continues to be a major public health problem in China with an estimated 1 million incident cases reported which alone contributed 11% to the global TB incident in 2010[2]. And the number of new TB cases in China ranks second in the world, despite that substantial progress has been made and the prevalence of TB had decreased from 215 per 100,000 in 1990 to 108 per 100,000 in 2010. 

TB is an airborne infectious disease with spatial autocorrelation in distribution[3,4]. The risks of TB transmission in an area are influenced by the epidemics of TB in neighboring areas, as shown by high-risk transmission areas cluster in some regions. Geographically, the burden of TB is highest in Asia and Africa globally. About 60% of reported TB cases occur in the South-East Asia and Western Pacific regions, and 24% in the African Region[1]. In China, the epidemic of TB is unevenly distributed within the country with higher prevalence in rural, especially in the relatively poor north and west areas. According to the 5^th^ national TB survey in 2010, the highest prevalences of active TB and sputum smear-positive (SS+) TB were observed in the west region, whereas the east region had the lowest prevalences[5]. The uneven distribution of the TB in the country highlights the importance of geographically tailed, pro-poor strategies in TB prevention and control in resources scarcity regions of China. 

New approaches such as spatial clustering analysis are powerful in identifying infectious disease epidemics. Spatial clustering analysis can detect spatial autocorrelation when the values of variables at nearby locations are not independent from each other[6]. The basic assumption of spatial autocorrelation is that biological processes such as speciation, extinction, dispersal or species interactions are distance-related[7]. Spatial autocorrelation may be seen as an opportunity for further analysis, as it provides information for inference of process from pattern such as geographic dispersal of TB[8,9]. A study conducted by Roza DL had used spatial analysis to identify the areas with elevated risk of TB and evaluate the influences of social vulnerability[10]. Factors affecting the distribution and pattern of TB, including TB notification rate and TB incidence rate etc., usually show spatial autocorrelation and obvious spatial heterogeneity[11], which is difficult for the traditional model to deal with[12]. The TB epidemic environments which have limited resources and tools to assist decision support need to take account of these limitations. However, spatial antocorrelation is one way to expore the relationship of factor among the neighboring areas. Additionally, there are few surveys developed for describing TB spatial distribution based on county-level nationally in China. 

The Chinese government has established a routine reporting system for notifiable infectious diseases in the 1950s[13,14]. In response to the outbreak of Severe Acute Respiratory Syndrome (SARS) in 2003, the system was switched from paper-based to web-based reporting. Currently this daily web based surveillance system covers 37 notifiable infectious diseases including TB and web-based reporting has been achieved in almost all the counties in China[15,16]. Every case of TB diagnosed is reported by county center for disease prevention and control (CDC) with information on age, sex, address, results of smear microscopy, diagnosis and other clinical information through the web based surveillance system from which notification data is generated. In China, county CDC takes the responsibility of case management and report within the county, and the case detection rate of TB in China has reached 89% according to the WHO[1].

The aim of this study is to use the spatial clustering analysis of county level TB notification data from 2005 to 2011 in China to determine the clustering areas of TB epidemic to provide evidences to local and national TB control program for strategy development and intensified intervention. 

## Materials and Methods

### 2.1: Setting

China has four administrative levels — town, county, prefecture and province from low to high in rural, corresponding to community, district, city and municipality in urban area[17]. The basic unit of reporting TB in the web based surveillance system is the county/district-level in rural and urban area. All counties/districts in mainland China were included in this study. The number of counties changed from 2862 in 2005 to 2856 in 2010 with the merging of some counties as part of administrative reorganization (Table 1)[18]. 

**Table 1:** Number of counties and population in China from 2005 to 2010.

**Variable**	**2005**	**2006**	**2007**	**2008**	**2009**	**2010**
No. of counties	2862	2860	2859	2859	2858	2856
No. of population (10 thousand)	130756	131448	132129	132802	133474	134091

### 2.2: Data source

County/district level TB notification data from 2005-2011 was extracted from the national surveillance system for notifiable infectious disease(Surveillance database). Defined by the results of smear microscopy and treatment history, two indicators were used in this study, i.e., notification rates of new SS+ TB and total TB (including SS+, sputum smear-negative (SS-), sputum smear not done, tuberculous pleurisy cases and extrapulmonary TB). The denominator of notification rate was the population in each county respectively from the national surveillance system. And the Cochran-Armitage Test was applied for tread test. All the TB data was geocoded and matched to the county-level polygon maps of the geographic information (Geographic database from China CDC) at a 1:1,000,000 scale as the layer’s attribute table by the same identified number. Furthermore, a county-level point layer containing information on latitudes and longitudes of central points for each county was created for the spatial-temporal analysis. 

### 2.3: Spatial clustering analysis

#### 2.3.1: Global spatial clustering analysis

Spatial autocorrelation which is an assessment of the correlation of a variable in reference to spatial location of the variable, is a match between location similarity and attribute similarity[19]. Global Moran’s *I*, a global test statistics for spatial autocorrelation, is based on cross-products for measuring attribute association[20]. The value of Global Moran’s *I* varies between -1 and 1. A higher positive Moran’s *I* indicates that values in neighboring positions tend to cluster, while a lower negative Moran’s *I* implies that higher and lower values are interspersed. When Moran’s *I* is near 0, there is no spatial clustering, meaning that the data are randomly distributed[21]. The data of this study were stored by ArcGIS 10 software to create a spatial database and the global spatial autocorrelation analysis was conducted using the package of Spatial Autocorrelation (Moran’s *I*) in ArcGIS 10. Furthermore, the reverse distance between two areas was considered as the conceptualization of spatial relationship, implying that nearby neighboring features had a larger influence on the computation for a target feature than that features far away[22]. Both *Z*-score and *p*-value were calculated to evaluate the significance of Global Moran’s *I*. In addition, Global Moran’s *I* scatter plot describing the trend and linear regression analysis was used to test the relationship between Global Moran’s *I* and time.

#### 2.3.2: Spatial-temporal clustering analysis

SaTScan version 9.1.1 using Kulldorf method of retrospective space-time analysis and Possion probability model, was applied to identify geographic areas and time period of potential clusters with high rates that had statistically significantly exceeded TB notification rates of nearby areas(*p*<0.05). SaTScan is a open source cluster software program developed by the National Cancer Institute (NCI) and other institutions. And the space-time scan statistics can be used for time-periodic surveillance, where the analysis is repeated every year[23]. Not only the geographical information, but also the time-periodic variable can be utilized by the space-time scan statistics to explore the possible spatial concentration and temporal persistence. In this study, the surveillance database for 7 years was collected from 2005 to 2011. TB cases notified in each county were used and recorded against the population in the same county which was assumed as the population in the Possion probability model. The space-time scan statistic creates an infinite number of discrete, cylindrical windows with a circular geographic base and with height corresponding to time. The base is defined exactly as for the purely spatial scan statistic, while the height reflects the time period of potential clusters. Each cylindrical window was evaluated as a possible TB space-time cluster[23]. Therefore, the space-time scan statistic that can use both the geographical information and the time-periodic variable simultaneously is more suitable for the database. The distribution and statistical significance of the space-time clusters was analyzed by means of Monte Carlo replication under the null hypothesis with the default 999 replications to ensure adequate power for defining clustering. Most likely clusters and secondary clusters were reported by SaTScan. The likelihood function is maximized over all window locations and sizes, and the one with the maximum likelihood constitutes the most likely cluster, which is least likely to have occurred by chance. Secondary clusters were detected in the same way as for the most likely cluster, by comparing the log likelihood ratio of secondary clusters in the real data set with the log likelihood ratios of the most likely cluster in the simulated data sets[23]. TB Clusters including most likely cluster and secondary clusters, were presented in this study.

## Results

### 3.1 TB notification rate

The study included all the counties/districts in mainland China across the 7-year study period. Total TB notification rate was decreasing from 2005 to 2011 (*z*=-123.19, *p*<0.01). Notification rate of new SS+ TB(*z*=-86.72, *p*<0.01) had the same trend with notification rate of total TB as well. (Table 2) The matched rates between the geographic database and the surveillance database were over 90% from 2005 to 2011. 

**Table 2:** Matching of surveillance and geographic databases and mean of three TB notification rates in China from 2005 to 2011.

	**Mean of notification rate(/100,000)**		**Matched rate among different databases (%)**	
**Year**	**new SS+**	**total TB**	**Geographic**	**Surveillance**
2005	40.5	86.2	91	95
2006	41.4	92.7	91	95
2007	40.2	94.1	91	95
2008	39.0	90.6	93	95
2009	37.1	85.2	93	95
2010	35.3	80.2	93	95
2011	31.9	78.1	93	94

### 3.2: Spatial clustering analysis

#### 3.2.1: Global spatial clustering analysis by Global Moran’s *I*

**Figure 1:**
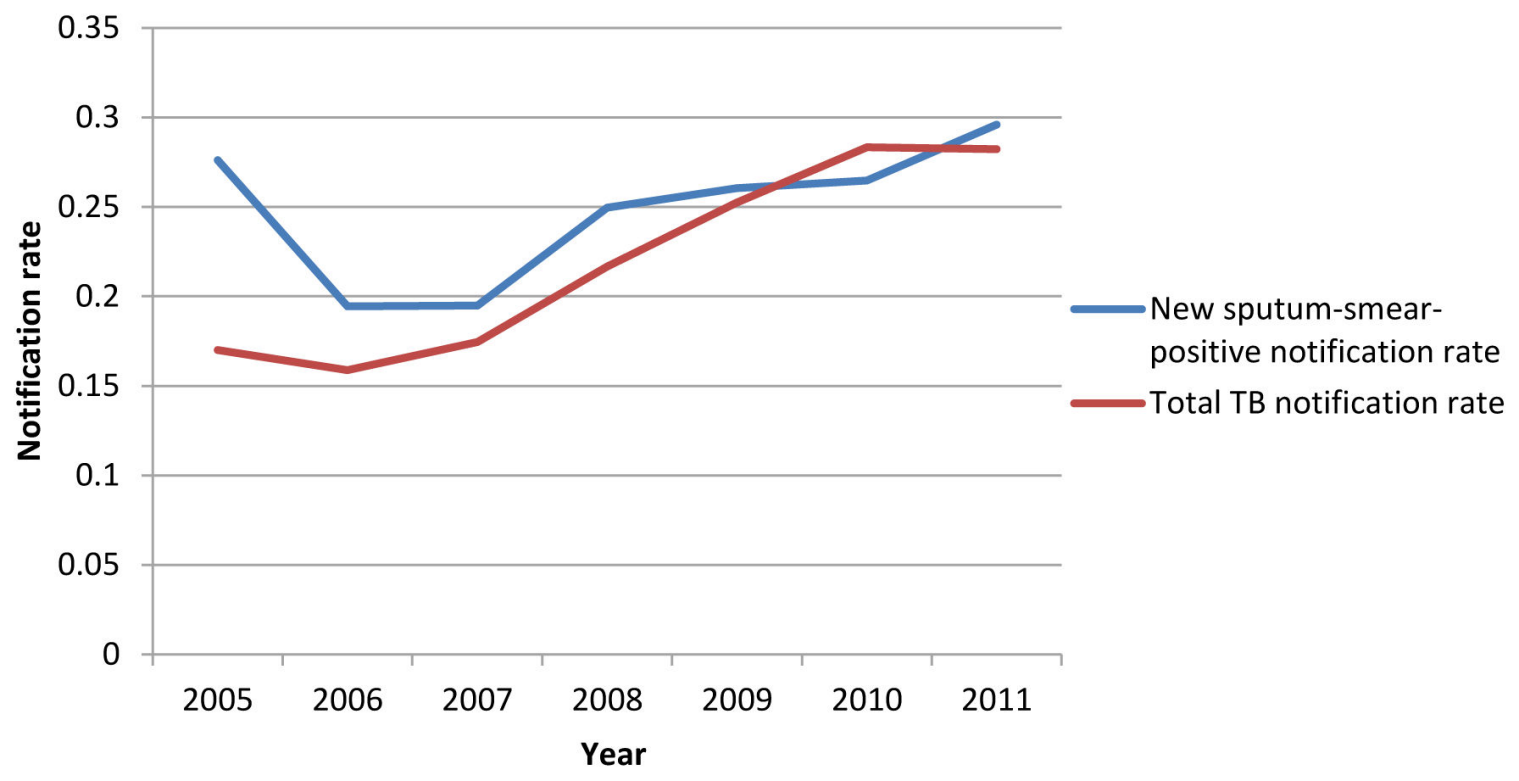
Trends of Moran’s I on two TB notification rates in China from 2005 to 2011.

In general, the significant global spatial autocorrelation existed in new SS+ notification rate and total TB notification rate from 2005 to 2011 respectively (*p* <0.01) (Table 3). The higher the absolute value of Global Moran’s *I* is, the stronger a spatial autocorrelation exists. The Global Moran’s *I* of total TB notification rate increased from 2005 to 2011 (*t*=6.87, *p*<0.01). It meant that TB cases had the trend with time to cluster in some areas globally. However, there were no trend on new SS+ notification rate in global spatial autocorrelation (*t*=1.29, *p*=0.25) (Figure 1). 

**Table 3:** Global spatial autocorrelation analysis of TB by Global Moran’s *I* in China from 2005 to 2011.

		**Global Moran's *I***		
**Indicators**	**Year**	**Moran's *I***	***Z*-score**	***p*-value**
New SS+ notification rate	2005	0.28	58.64	<0.01
	2006	0.19	60.79	<0.01
	2007	0.20	40.65	<0.01
	2008	0.25	57.50	<0.01
	2009	0.26	59.51	<0.01
	2010	0.26	60.76	<0.01
	2011	0.30	67.83	<0.01
Total TB notification rate	2005	0.17	36.38	<0.01
	2006	0.16	49.93	<0.01
	2007	0.17	36.81	<0.01
	2008	0.22	50.49	<0.01
	2009	0.25	57.82	<0.01
	2010	0.28	65.08	<0.01
	2011	0.28	64.77	<0.01

#### 3.2.2: Spatial-temporal clustering analysis by SaTScan

**Figure 2:**
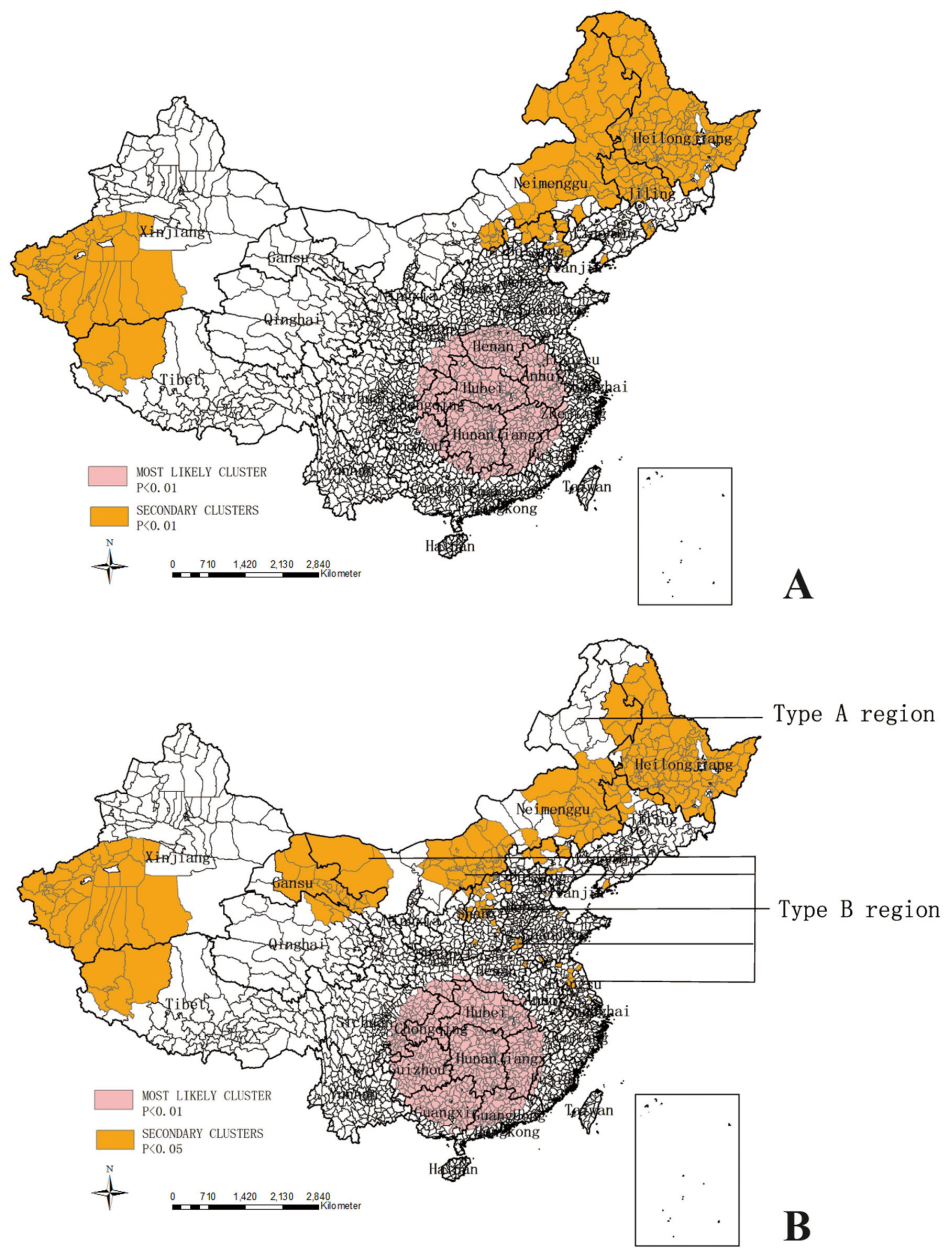
SaTScan analysis on new SS+ notification rate (A) and total TB notification rate (B) from 2005 to 2011.

Spatial-temporal clustering analysis by SaTScan indicated that the most likely clusters with high rates of new SS+ notification and total TB notification respectively which had similar clustering size, located spatially in the south-central regions of China from 2006 to 2008 (Table 4, Figure 2). However, the secondary clusters of total TB notification rate (Figure 2-B) had different distribution from that of new SS+ notification rate (Figure 2-A). The secondary clusters of new SS+ notification rate was in two extreme regions: (1) northeast of China including Heilongjiang province and the parts of Inner Mongolia and Jilin province, (2) west of China including the west parts of Tibet and Xinjiang Autonomous Region. Besides, the secondary clusters of total TB notification rate had two more large clustering centers in Inner Mongolia, Gangsu and Qinghai province and some small clustering centers in Shanxi, Henan, Hebei and Jiangsu province.

**Table 4:** Significant high-rate TB Clusters detected by SaTScan in China from 2005 to 2011.

		**Indicators**	
**Category of clustering**	**Index**	**New SS+ notification rate**	**Total TB notification rate**
Most likely cluster	Number of clustering centers	1	1
	Coverage of clustering centers (counties)	639	651
	Log likelihood ratio	16072	36820
	*p*	<0.01	<0.01
Secondary clusters	Number of clustering centers	24	62
	Coverage of clustering centers (counties)	263	340
	Log likelihood ratio	20~9145	14~27430
	*p*	<0.01	<0.05

## Discussion

Spatial-temporal analysis by SaTScan such as described in this study identified TB space-time clusters. The present analysis underscored the first and secondary clusters where people bear the persistant burden of TB, and detected 3-year persistence of excess TB burden with the highest relative risk from 2006 to 2008. Simultaneously, most clusters for new SS+ notification rate mentioned above overlapped with the clusters for total TB notification rate. The most likely and secondary clusters which overlapped among Figure 2-A and B had higher TB burden and more risks of TB transmission and these should be the important areas for TB control nationally. Interventions should be targeted for these overlapped clusters and surrounding areas. 

New SS+ TB as the numerator of new SS+ notification rate is one part of Total TB as the numerator of Total TB notification rate. Total TB includes SS+ TB, SS- TB, sputum smear not done, tuberculous pleurisy cases and extrapulmonary TB. It is widely believed that smear positive patients with TB are more infectious than smear negative patients. Thus new SS+ notification rate is one indicator of TB transmission risk and Total TB notification rate is one indicator of TB burden. Because Type A region in Figure 2 was under the coverage of clusters for new SS+ notification rate but not under the coverage of clusters for total TB notification rate, Type A region had less TB burden than the neighboring areas on SS- cases, sputum smear not done, tuberculous pleurisy cases and extrapulmonary TB, but more risks of TB transmission. This may be explained by (1) Type A region is mountainousand has facilities with lesser techniques of TB diagnosis. Many TB patients with atypical symptoms of TB in Type A region had to go to the big hospitals with high techniques for further diagnosis which are located in other big cities. As such TB patients then were reported by those big hospitals (2). this area is characterized by low temperatures and hence the people live in the very close proximity which increases the risk of TB transmission among the inhabitants.

In addition, two more large clusters were identified in Inner Mongolia, Gangsu and Qinghai Provinces and several smaller clusters in Hebei, Shanxi, Henan and Jiangsu Provinces (Figure 2-B). These clusters were in the relatively poor provinces with less techinques of TB diagnosis and treatment. Hohhot and Taiyuan cities as the provincial capitals of Inner Mongolia and Shanxi provinces respectively have designed TB facilities with higher techniques for TB diagnosis and treatment, clustered more cases which can not be diagnosed in local hospitals with lesser techniques in other prefecture cities or counties. As an indicator of TB burden, total TB notification rate lumps together SS- cases, sputum smear not done, tuberculous pleurisy cases and extrapulmonary TB besides SS+, this was associated with more clustering as shown in Figure 2-B. This patient flow from prefecture city or county to provincial capital could result in the difference of clusters between Figure 2-B and A. Furthermore, the clustering of global spatial autocorrelation showed positive trend with the time globally (*t*=6.87, *p*<0.01). It meant that the spatial distribution of total TB notification rate became more and more clustered as time went by. And as one challenge in China, the patient flow from prefecture city or county to provincial capital that will continue for better diagnosis and treatment in a few years, is one of the factors to influence the clustering of TB. 

Global spatial clustering analysis was performed to evaluate whether the global spatial clustering exist globally and Global Moran’s *I* is a gloabal index for the spatial cluatering analysis. Furthermore, the trend test of Moran’s *I* indicated whether the clusters would aggregate with time. However, the clustering areas can not be detected by Global spatial clustering analysis. Therefore, Spatial-temporal clustering analysis by SaTScan was utilized. SaTScan would still be concentrated in the geographical clustering areas. Simultaneously, it would be extended in a third dimension reflecting the population size as it changes over time. Although the results of global spatial autocorrelation on new SS+ notification rate didn’t indicate significant temporal trend, the statistical analysis of Global Moran’s *I* every year was significant and the clusters were detected by SaTScan in the south-central, northeast and west regions of China. As patients with sputum smear–negative TB are less infectious than patients with sputum smear–positive TB[24], this may suggest that close contacts of patients with positive smear were more likely to develop TB disease[25]. Thus, areas of clusters for new SS+ notification rate had more risks of TB transmission than the other areas. But these clusters didn’t have the trend to aggregate year by year due to insignificant statistics of the trend test for Moran’s *I*. Thus, it was suggested that these areas of clusters for new SS+ notification rate with high risk of TB transmission in population should allow prioritization of focused infection control interventions. 

Spatial analysis has been used to detect the distribution and pattern of various infectious diseases and non-communicable diseases by GIS, SaTScan and other softwares, and acquired meaningful results[26,27]. Researchers can detect patterns and relationships in the data based on geography through spatial analysis. Because these clusters carry a disproportional burden of excess TB, the results are helpful for TB control activities that direct public health action and guide interventions. Clusters including most likely clusters and secondary clusters, were presented in this study prioritized for public health action according to the statistical analysis[28]. Thus, to accomplish the target of descreasing TB cases, it will be necessary to (1) implement more effective and stronger measures to control TB transmission in the clusters, especially in the clustering areas of new SS+ notification rate; (2) intensify the case-finding, not only of SS+ cases but also of SS-, sputum smear not done, tuberculous pleurisy cases and extrapulmonary TB in the clusters of total TB notification rate and other cities or counties in the same provinces (Figure 2-B); (3) enhance treatment and management of TB cases under programmatic conditions in all clusters. These techniques applied in this study contribute to TB control and prevention in China, and then the resouces of TB control can be rearranged according the analysis. 

The identification of TB risk areas using surveillance data based on geographic community is a relatively inexpensive undertaking[29]. However, a handful focused on TB have been conducted[30], especially in China. In addition, some previous studies focused on small geographical areas and lacked national coverage. This study that analyzed the basic reporting unit, county-level at the national wide scale, is therefore the first to analyze risk at county level nationally. The national representativeness is better than the other studies of the sample and the bias decreased, due to subject increasing to the whole country for spatial analysis. 

Though our study demonstrated the usefulness of spatial and temporal clustering analyzing by ArcGIS and SaTScan in China, it still had some limitations. Firstly, the data was analysed at county-level - the basic unit of surveillance system in China - which is not the smallest unit of administrative regionalization. Some towns with insignificant clustering might be covered by the clusters based on the county-level. Secondly, while the matched rates were over 90% between Geographic database and Surveillance database, the unmatched subjects might influence the spatial analysis. Thirdly, potential risk factors that could be associated with the clustering were not assessed in this study. The effect of risk factors to the clustering of TB notification rates including natural and social-economic factors, could be assessed and adjusted in the futher studies. 

Disease clusters detection in space and/or time may play a great role in public health policy making. Systemic utilization of cluster detection techniques for regular surveillance of TB may help the TB program in disease control activities[30]. This study had identified the significant space-time clusters of TB. The integrated analysis of different clusters of 2 notification rates may be used to guide the provision and optimization of TB control strategies in China. And the good cost-effectiveness would be achieved if more resource and effective measures for TB control were provided and implemented in these clusters. The experience and methods of TB clusters detection and analysis in this study will benefit to other high TB burden countries as well.
